# Post-Exercise Skeletal Muscle Glycogen Related to Plasma Cytokines and Muscle IL-6 Protein Content, but not Muscle Cytokine mRNA Expression

**DOI:** 10.3389/fnut.2015.00027

**Published:** 2015-09-09

**Authors:** David C. Nieman, Kevin A. Zwetsloot, Mary Pat Meaney, Dominic D. Lomiwes, Suzanne M. Hurst, Roger D. Hurst

**Affiliations:** ^1^Appalachian State University, Kannapolis, NC, USA; ^2^The New Zealand Institute for Plant and Food Research Ltd., Palmerston North, New Zealand

**Keywords:** running, interleukin-6, interleukin-8, monocyte chemoattractant protein-1, chemokines

## Abstract

**Objectives:**

The purpose of this study was to correlate post-exercise muscle glycogen levels with changes in plasma cytokine, and muscle mRNA cytokine expression and protein content.

**Methods:**

Twenty-four male runners (age 36.5 ± 1.8 years, VO_2max_ 60.0 ± 1.5 mL⋅kg^−1^ ⋅ min^−1^) ran twice (separated by 4 weeks) on treadmills to exhaustion at 70% VO_2max_ (average time and distance of 2.24 ± 0.09 h and 24.9 ± 1.1 km). Muscle biopsies from the vastus lateralis and blood samples were collected before and after each run, with IL-6, IL-8, and MCP-1 measured in muscle (mRNA and protein) and plasma. Data from the two runs were averaged.

**Results:**

Participants experienced a 35.3 ± 4.2% decrease (*P* < 0.001) in skeletal muscle glycogen content (67.5 ± 2.8 to 44.3 ± 3.7 mmol⋅kg^−1^ wet weight). Muscle mRNA expression for IL-6, IL-8, and MCP-1 increased 7.34 ± 0.90-, 13.9 ± 2.3-, and 4.10 ± 0.60-fold, respectively (all, *P* < 0.001). Skeletal muscle IL-6, IL-8, and MCP-1 protein content increased 35.8 ± 10.6, 80.6 ± 12.1, and 105 ± 17.9%, respectively (all, *P* ≤ 0.005). Plasma IL-6, IL-8, and MCP-1 increased 47.1 ± 10.0-, 2.6 ± 0.3-, and 1.6 ± 0.1-fold, respectively (all, *P* < 0.001). Post-exercise muscle glycogen concentrations were negatively correlated with run time to exhaustion (*r* = −0.70, *P* < 0.001), and changes in muscle IL-6 protein content (*r* = −0.44, *P* = 0.049), plasma IL-6 (*r* = −0.72, *P* < 0.001), IL-8 (*r* = −0.60, *P* = 0.002), and MCP-1 (*r* = −0.589, *P* = 0.002), but not with changes in muscle IL-8 and MCP-1 protein content, or muscle mRNA expression for IL-6, IL-8, and MCP-1.

**Conclusion:**

Prolonged and intensive running increased muscle mRNA expression, muscle protein content, and plasma levels for IL-6, IL-8, and MCP-1, and post-run muscle glycogen levels were most strongly related to plasma cytokine levels.

## Introduction

Cytokines are a broad category of small proteins that are important in cell signaling, act through receptors, and affect the behavior of other cells. Cytokines are released by a range of immune cells, endothelial cells, adipocytes, muscle cells, and other cells, and include chemokines, interferons, interleukins, lymphokines, and tumor necrosis factor (1–3). Considerable attention has focused on the potential for regular exercise to counteract a range of disease states by stimulating cytokine production and release (2, 3).

The six cytokines with the greatest fold increase in plasma during prolonged and intensive exercise include IL-6, IL-10, granulocyte colony stimulating factor (G-CSF), IL-8, interleukin-1 receptor antagonist (IL-1ra), and monocyte chemoattract protein-1 (MCP-1) (4–6). These cytokines exert anti-inflammatory and chemotactic influences, and are produced by multiple cell types both within and outside the immune system and skeletal muscle tissue (1, 3, 7). Post-exercise muscle biopsy samples contain muscle cells that can produce and release IL-6 and MCP-1, resident macrophages (IL-6, IL-10, IL-8, MCP-1, and G-CSF), and endothelial/epithelial cells (IL-8, MCP-1, IL-1ra, and G-CSF) (1, 4, 8–12). Blood monocytes and lymphocytes can contribute to elevated plasma cytokine levels following exercise by producing IL-10, IL-1ra, and IL-8 (4). The acute, large exercise-induced increases in IL-6 and other cytokines orchestrate anti-inflammatory influences, augment lipolysis, stimulate chemotaxis, improve insulin sensitivity, serve as signaling agents for training adaptations and tissue repair, and function in many other roles related to health (1–3, 8).

The primary signaling mechanisms for inducing cytokine gene expression and production during intensive exercise are still being investigated, with a focus on muscle damage and metabolic demands, including glycogen depletion and total exercise workload (1, 8, 9, 11, 13–20). Post-exercise cytokine gene expression and secretion can be modified by other forms of physiological stress (e.g., heat, mode of exercise, age) (19, 21–23) medications (e.g., ibuprofen) (5), and nutritional factors (carbohydrate availability, high vitamin E intake, free fatty acid availability, and polyphenol supplementation) (11, 16, 24, 25). Carbohydrate ingestion before and during intensive and prolonged exercise, especially in overnight fasted endurance athletes, decreases skeletal muscle mRNA expression for IL-6 and IL-8, and lowers post-exercise plasma levels for IL-6, IL-8, IL-1ra, and IL-10 (11).

In response to published reports that carbohydrate ingestion during prolonged exercise attenuated the plasma IL-6 response, Steensberg et al. (26) hypothesized that a link may exist between low muscle glycogen content and IL-6 production during exercise. Using a two-legged knee-extensor exercise design, IL-6 mRNA levels and IL-6 release were shown to be augmented in the ~40% glycogen-depleted leg compared with the control leg in seven men who exercised for 5 h. Keller et al. (27) showed that muscle IL-6 mRNA and transcriptional activity were enhanced when six male participants engaged in 3 h of two-legged knee-extensor exercise with low muscle glycogen levels. Chan et al. (13) reported that exercising (60 min cycling, ~70% VO_2max_) in a glycogen-depleted state increased muscle IL-6 and IL-8 mRNA expression, with slightly higher plasma IL-6 but not IL-8 levels. Other studies have not been able to show that variance in muscle glycogen depletion with exercise is related to muscle IL-6 or IL-8 mRNA expression (11, 14, 15). Helge et al. (15) showed that IL-6 release is markedly higher from the exercising arms than the legs during whole-body exercise, and is not related to muscle glycogen utilization. Thus, the role of glycogen availability in the contracting muscle as a primary signaling mechanism for cytokine mRNA expression and release into the circulation is unclear, and interpretation has been made more difficult through research designs utilizing small subject numbers and modest exercise workloads (14–17).

The purpose of this study was to relate post-exercise muscle glycogen levels with muscle mRNA IL-6, IL-8, and MCP-1 expression and protein content, and change in plasma cytokine levels in 24 male runners who ran twice on treadmills to exhaustion at ~70% VO_2max_. Muscle biopsies from the vastus lateralis and blood samples were collected before and after each run, and data from the two runs were averaged to strengthen data quality, reduce random measurement error, and provide better estimates of the “true” value for individual participants (28).

## Materials and Methods

### Participants

Subject recruitment was conducted via direct messaging to runners and running clubs in the Charlotte, NC, USA, metropolitan area. Participants included 24 male runners (ages 27–49 years) who regularly competed in long-distance road races and were capable of running at ~70% on treadmills to exhaustion. During the study, participants consented to train normally, maintain weight, and avoid the use of large-dose vitamin and mineral supplements (above 100% Daily Value), and all herbal supplements and medications (in particular, non-steroidal anti-inflammatory drugs) for the 4-week period separating the two lab sessions. Participants signed informed consent forms and study procedures were approved by the Institutional Review Board at Appalachian State University (ASU).

### Baseline testing

Two weeks prior to the first running trial in the ASU Human Performance Laboratory, participants were tested for VO_2max_ during a graded, treadmill test with the Cosmed Fitmate metabolic system (Cosmed, Rome, Italy) (29). Body composition was measured with the Bod Pod body composition analyzer (Life Measurement, Concord, CA, USA). Demographic and training histories were acquired with questionnaires.

### Running trials

During the 3-day period prior to each running trial (separated by 4 weeks), participants followed a moderate-carbohydrate dietary regimen (~55% kcal as carbohydrates) by choosing foods from a list provided by the investigative team. A standardized meal consisting of Boost Plus (Néstle Nutrition, Slorham Park, NJ, USA) was ingested at 12:00 p.m., with energy intake adjusted to 5 kcal⋅kg^−1^ body weight. Boost Plus is a nutritionally complete, high-energy oral supplement with an energy density of 6.4 kJ⋅mL^−1^ (1.52 kcal⋅mL^−1^) and 15% of energy as protein, 35% as fat, and 50% as carbohydrate, and 24 vitamins and minerals. Participants reported to the lab at 2:15 p.m. and provided blood and muscle biopsy samples. At 3:00 p.m., participants ran on laboratory treadmills with the speed set at 70% of VO_2max_, and drank water *ad libitum*, without ingestion of any other beverages or food. Participants ran as long as possible until exhaustion, defined as the inability of the subject to continue running at 70% VO_2max_ despite verbal urging from the laboratory staff. Metabolic measures from the Cosmed Fitmate metabolic system and the rating of perceived exertion (RPE) were taken at 15 min, and then every 60 min during the running bout to verify that the appropriate intensity was maintained. Blood and muscle samples were taken again immediately following exercise. These procedures were repeated 4 weeks later, with runners following the exact same schedule (including same time and day of the week), and running at the same treadmill speed to exhaustion.

### Cytokine analysis

Total plasma concentrations of three cytokines [monocyte chemoattractant protein-1 (MCP-1), IL-6, and IL-8] were determined using an electrochemiluminescence-based solid-phase sandwich immunoassay (Meso Scale Discovery, Gaithersburg, MD, USA). All samples and provided standards were analyzed in duplicate, and the intra-assay CV ranged from 1.7 to 7.5% and the inter-assay CV 2.4–9.6% for all cytokines measured. Pre- and post-exercise samples for the cytokines were analyzed on the same assay plate to decrease inter-kit assay variability.

### Stress hormone analysis

Serum cortisol was measured with an electrochemiluminescence immunoassay (ECLIA), and plasma epinephrine with high-pressure liquid chromatography (HPLC) and electrochemical (EC) detection through a commercial lab (LabCorp, Burlington, NC, USA).

### Muscle biopsy procedures and glycogen analysis

Pre- and post-exercise muscle biopsy samples were acquired from the vastus lateralis on the same leg ~2 cm apart using procedures previously described (30). During the second running trial, the same procedures were followed, but on the opposite leg. Local anesthesia (1% xylocaine, Hospira, Inc., Lake Forest, IL, USA) was injected subcutaneously. After a small incision, a muscle biopsy sample was obtained using the suction-modified percutaneous needle biopsy procedure (30). Muscle was trimmed of connective tissue and fat and immediately frozen in liquid nitrogen. Samples were stored at −80°C until subsequent analysis. A glycogen assay kit (Catalog #MAK016, Sigma-Aldrich, St. Louis, MO, USA) was used to determine the concentration of glycogen in vastus lateralis muscle homogenates. In this coupled enzyme assay, glucoamylase hydrolyzed glycogen to glucose, and then the glucose was oxidized to yield a product that reacted with a probe to generate a color detectable with a microplate reader (Synergy H1 Hybrid Reader, BioTek Instruments, Inc., Winooski, VT, USA) at 570 nm.

### Muscle mRNA measurements

Total RNA extraction of vastus lateralis biopsies was conducted using an RNeasy^®^ Fibrous Tissue Mini Kit (Qiagen, Limburg, Netherlands) according to the manufacturer’s instructions. Frozen muscle biopsies were homogenized in RTL-β-mercaptoethanol Buffer with a Precellys homogenizer (Bertin Technologies, Montigny-le-Bretonneux, France). An aliquot of RNA from all samples were run on 1.5 denaturing agarose gels stained with ethidium bromide to confirm RNA integrity. Additionally, RNA quality was further verified spectrophotometrically using the A_260_/A_280_ ratio which was determined using a nanodrop. Reverse transcription of RNA into cDNA was conducted using a High Capacity cDNA Reverse Transcription Kit (Applied Biosystems, Foster City, CA, USA) as per manufacturer’s instructions. Reverse transcription was performed using a GeneAmp^®^ PCR System 9700 (Applied Biosystems). cDNA was quantified using a nanodrop and aliquots were stored at −20°C until Real Time-Polymerase Chain Reaction (RT-PCR) analysis.

Quantitative RT-PCR analysis of messenger RNA (mRNA) was conducted under pre-optimized TaqMan Gene Expression Assays (Applied Biosystems) using gene specific FAM dye-labeled primers for IL-6 (Cat. No. 4453320), IL-8 (Cat. No. 4369514), MCP-1 (Cat. No. 4331182), and beta-2-microglobulin (B2M) (Cat. No. 4351370). Samples and reagents were loaded in a MicroAmP Fast-Optical 96 Well Reaction Plate and run in quadruplicate. Plates were analyzed on a 7500 Fast RT-PCR System (Applied Biosystems). Relative mRNA levels were determined using the ΔΔ*C*_t_ calculations with B2M serving as the housekeeping gene (31). Changes in cytokine expression post-exercise were normalized to pre-exercise levels.

### Analysis of skeletal muscle inflammatory cytokine protein concentrations

Skeletal muscle biopsy samples were analyzed for the inflammatory cytokines IL-6, IL-8, and MCP-1 with a magnetic bead-based multiplex assay using the MAGPIX instrument and xPONENT^®^ analysis software (Luminex, Austin, TX, USA). Briefly, ~30 mg of each skeletal muscle tissue biopsy sample was homogenized in lysis buffer (Millipore, Billerica, MA, USA; #43-040) with AEBSF protease inhibitor added (Millipore; #101500). Homogenates were cleared by centrifugation at 14,000 ×* g* and the protein concentration of the supernatant was determined using a BCA protein assay kit (Pierce ThermoFisher, Rockford, IL, USA). Next, 30 μg of muscle protein was added to each well (in duplicate) and the concentration of each cytokine was measured using the MILLIPLEX^®^ MAP assay kit (Millipore, #HCYTOMAG-60K) according to manufacturer’s specifications. The lower limit of detection and inter-assay% coefficient of variability (% CV) for this panel of analytes was: IL-6 = 0.17 pg⋅mL^−1^ (4.2% CV); IL-8 = 0.18 pg⋅mL^−1^ (5.9% CV); and MCP-1 = 0.30 pg⋅mL^−1^ (3.0% CV). The intra-assay% CVs were IL-6 = 13.6%, IL-8 = 8.9%, and MCP-1 = 3.7%.

### Statistical analysis

Data are expressed as mean ± SE. Dependent *t*-tests were used to test change (pre- to post-exercise), with the level of significance set at *P* < 0.01. The Pearson product-moment correlation coefficient was used as a measure of the linear correlation between two variables.

## Results

Participants included 24 trained male runners (ages 22–55 years) who successfully adhered to all aspects of the study design (see subject characteristics in Table 1). Run distances to exhaustion and post-exercise muscle glycogen content for the two run trials were significantly correlated (*r* = 0.74, *P* < 0.001; *r* = 0.67, *P* < 0.001, respectively), as were between trial post-run plasma cytokines levels (*r* = 0.475, 0.545, and 0.584 for plasma IL-6, IL-8, and MCP-1, respectively, all, *P* < 0.05). Between trial correlations for muscle cytokine mRNA and protein levels were non-significant. Metabolic and performance data from the average of the two run-to-exhaustion trials are summarized in Table 2. The runners were able to maintain an average intensity of 69.2 ± 1.3% VO_2max_ for 2.24 ± 0.09 h and 24.8 ± 1.1 km. The RPE was 17.8 ± 0.2 at the end of the running trials.

**Table 1:** **Subject characteristics (*N* = 24)**.

Variable	Mean ± SE
Age (years)	36.5 ± 1.8
Height (m)	1.78 ± 0.01
Weight (kg)	77.3 ± 1.9
Body fat (%)	15.0 ± 1.0
VO_2max_ (mL⋅kg^−1^⋅min^−1^)	60.0 ± 1.5
HR_max_ (beats⋅min^−1^)	185 ± 2.3

**Table 2:** **Average metabolic and performance data for the two runs to exhaustion by trained runners**.

Variable	Mean ± SE
Time (h)	2.24 ± 0.09
Distance (km)	24.8 ± 1.1
VO_2_ (mL⋅kg^−1^⋅min^−1^)	41.3 ± 0.8
VO_2_ (% VO_2max_)	69.2 ± 1.3
HR (beats⋅min^−1^)	158 ± 2.0
% HR_max_	85.2 ± 0.7
Ventilation (L ⋅min^−1^)	77.2 ± 2.0
RPE	13.9 ± 0.2

*VO_2_, volume of oxygen consumed; HR, heart rate; RPE, rating of perceived exertion*.

**Figure 1:**
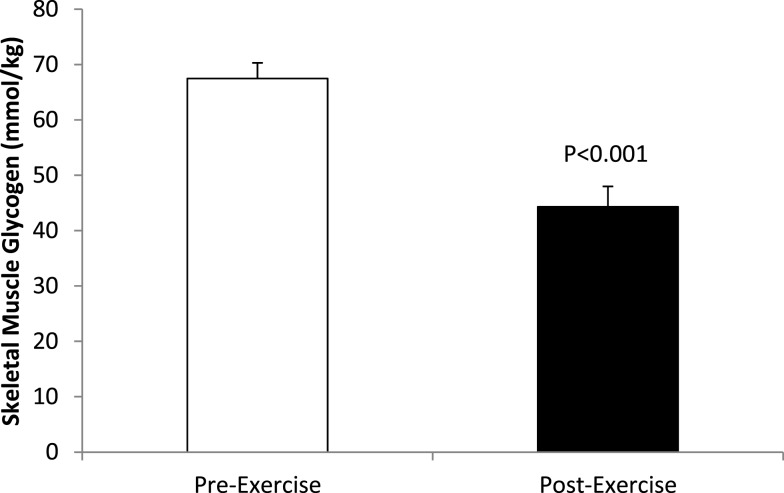
**Pre-and post-exercise skeletal muscle glycogen levels in *N* = 24 runners**.

Skeletal muscle glycogen sampled from the vastus lateralis decreased 35.3 ± 4.2% (23.3 ± 2.4 mmol⋅kg^−1^) (*P* < 0.001), with post-run values averaging 44.3 ± 3.7 mmol⋅kg^−1^ (range of 7.9–76.7 mmol⋅kg^−1^) (Figure 1).

**Figure 2:**
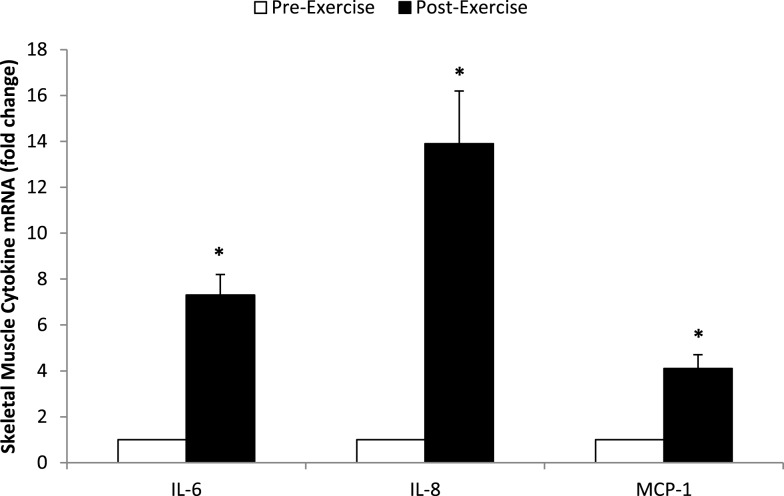
**Fold change in skeletal muscle mRNA expression for IL-6, IL-8, and MCP-1 in *N* = 24 runners**. *P < 0.001, change pre- to post-exercise.

**Figure 3:**
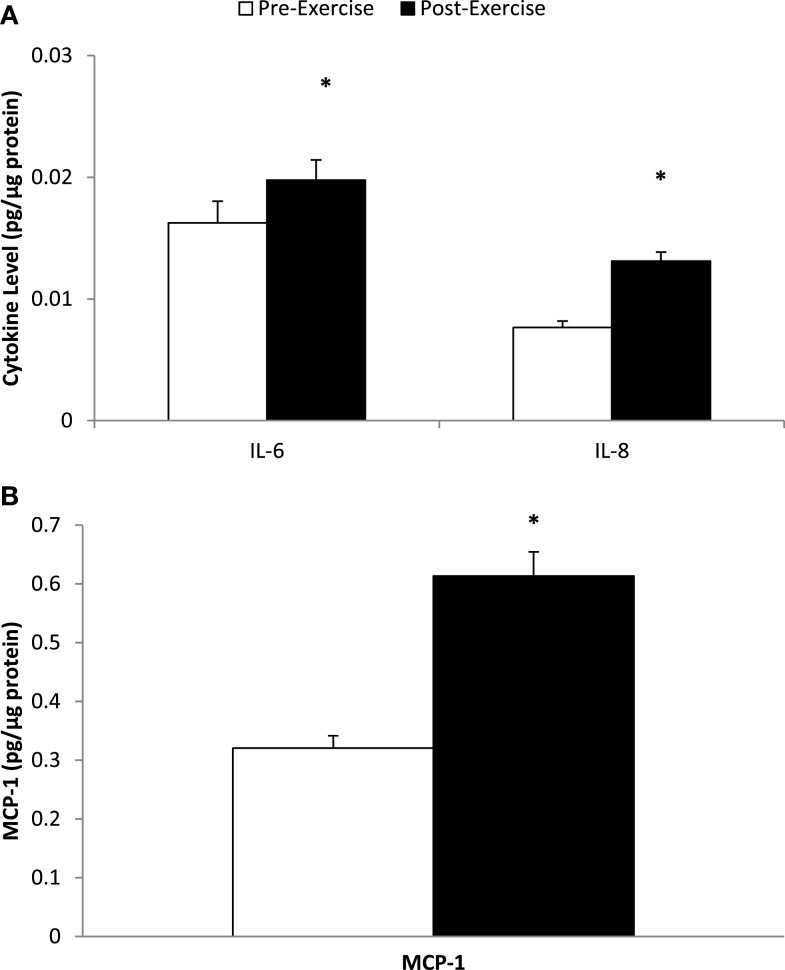
**Skeletal muscle cytokine protein content for (A) IL-6 and IL-8, and (B) MCP-1**. *P ≤ 0.005, change pre- to post-exercise.

Muscle mRNA expression for IL-6, IL-8, and MCP-1 increased 7.34 ± 0.90-, 13.9 ± 2.3-, and 4.10 ± 0.60-fold, respectively (all, *P* < 0.001) (Figure 2). Skeletal muscle cytokine protein content increased 35.8 ± 10.6% (*P* = 0.005) and 80.6 ± 12.1% (*P* < 0.001) for IL-6 and IL-8, respectively (Figure 3A), and 105 ± 17.9% (*P* < 0.001) for MCP-1 (Figure 3B).

Plasma cytokine and stress hormone data are summarized in Table 3. Plasma IL-6, IL-8, and MCP-1 increased 47.1 ± 10.0-, 2.6 ± 0.3-, and 1.6 ± 0.1-fold, respectively (all, *P* < 0.001). Serum cortisol and plasma epinephrine were elevated post-exercise 99.1 ± 14.42 and 147 ± 18.3%, respectively. No significant correlations were found between pre-to-post changes for each of the cytokines with changes in serum cortisol and plasma epinephrine (all, *P* > 0.05). Running distance to exhaustion was significantly correlated with change in plasma IL-6 (*r* = 0.503, *P* = 0.012), IL-8 (*r* = 0.665, *P* < 0.001), and MCP-1 (*r* = 0.735, *P* < 0.001).

**Table 3:** **Exercise effects on plasma cytokines and stress hormones**.

Variable	Pre-exercise	Post-exercise
Plasma IL-6 (pg⋅mL^−1^)	0.68 ± 0.07	21.1 ± 2.5*
Plasma IL-8 (pg⋅mL^−1^)	4.24 ± 0.24	14.9 ± 1.0*
Plasma MCP-1 (pg⋅mL^−1^)	100 ± 3.1	264 ± 13.7*
Serum cortisol (nmol⋅L^−1^)	272 ± 11.0	513 ± 33.1*
Plasma epinephrine (pmol⋅L^−1^)	390 ± 26.2	912 ± 66.6*

*P < 0.001 versus pre-exercise.

*IL, interleukin; MCP-1, monocyte chemoattractant protein-1*.

**Figure 4:**
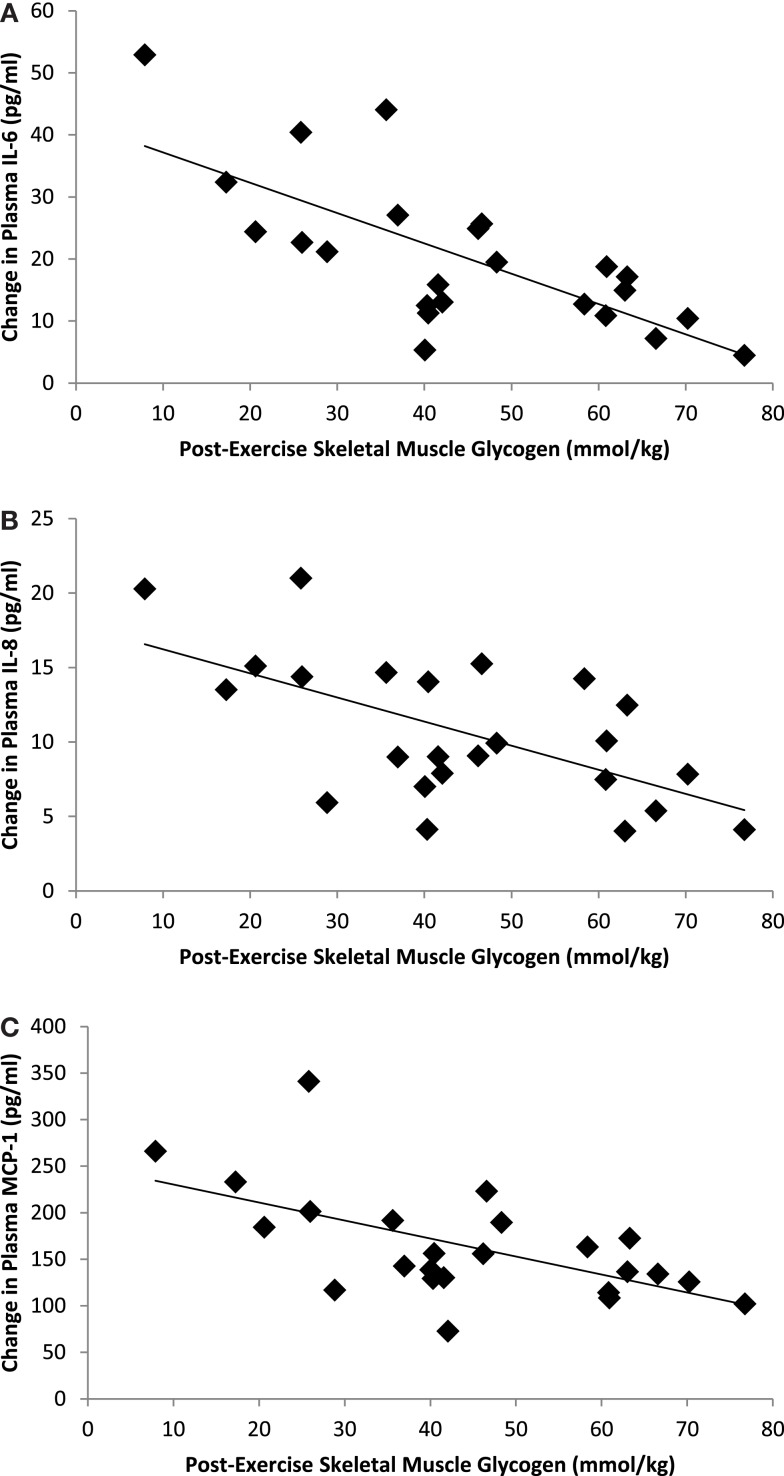
**Correlations between post-exercise skeletal muscle glycogen content and change in plasma (A) IL-6 (*r* = −0.718, *P* < 0.001), (B) IL-8 (*r* = −0.604, *P* = 0.002), and (C) MCP-1 (*r* = −0.589, *P* = 0.002)**.

**Figure 5:**
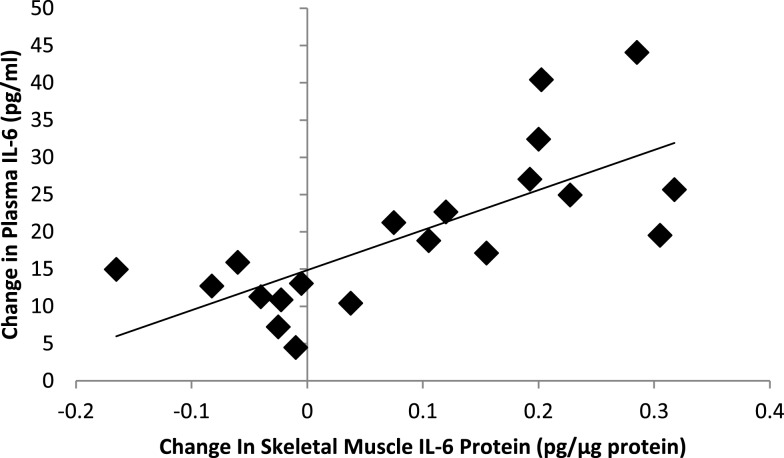
**Correlation between exercise-induced changes in skeletal muscle and plasma IL-6 levels (*r* = 0.73, *P* < 0.001)**.

Correlation statistics were calculated for pre-run, post-run, and absolute change in muscle glycogen with the outcome measures included in this study, and the strongest correlations (when significant) were found when using post-run glycogen values. Significant inverse correlations were measured between post-run glycogen levels and running time (*r* = −0.70, *P* < 0.001) and distance (*r* = −0.609, *P* = 0.002), but not with muscle mRNA expression for MCP-1 (*r* = −0.032, *P* = 0.882), IL-8 (*r* = −0.349, *P* = 0.095), or IL-6 (*r* = −0.211, *P* = 0.321). Post-run glycogen levels were negatively related to muscle IL-6 protein content (*r* = −0.55, *P* = 0.049), but not significantly to muscle IL-8 (*r* = −0.36, *P* = 0.120) or MCP-1 (*r* = −0.33, *P* = 0.160) protein content. Post-run glycogen levels were negatively related to change in plasma IL-6 (*r* = −0.718, *P* < 0.001), IL-8 (*r* = −0.604, *P* = 0.002), and MCP-1 (*r* = −0.589, *P* = 0.002) (Figure 4), but not to change in serum cortisol (*r* = 0.011, *P* = 0.959) or plasma epinephrine (*r* = 0.15, *P* = 0.478) (correlation figures not shown). Changes in muscle and plasma IL-6 levels were significantly related (*r* = 0.73, *P* < 0.001) (Figure 5), but not as strongly for muscle and plasma IL-8 (*r* = 0.40, *P* = 0.080) or MCP-1 (*r* = 0.413, *P* = 0.071) (correlation figures not shown). Changes in serum cortisol and plasma epinephrine were not significantly related to changes in muscle IL-6, IL-8, or MCP-1 mRNA or protein levels (all, *P* > 0.05) (correlation figures not shown).

## Discussion

As expected, these data from 24 male endurance athletes who ran twice on treadmills to exhaustion at ~70% VO_2max_ showed increases in muscle IL-6, IL-8, and MCP-1 mRNA and protein content, and corresponding increases in plasma levels for these three cytokines. This study focused on the linkage between muscle glycogen availability and cytokine mRNA expression and production, and showed that post-run muscle glycogen content was negatively related to plasma but not muscle mRNA levels for IL-6, IL-8, and MCP-1. No significant correlations were found between exercise-induced changes in stress hormones and plasma or muscle cytokines (both mRNA and protein levels), similar to results previously reported by our research group (10, 11).

The correlation between low muscle glycogen content and enhanced muscle cytokine mRNA expression is not a consistent finding, and depends on the study design, with the strongest support demonstrated when participants initiate exercise with low muscle glycogen levels (13, 26, 27). When participants engage in prolonged, intensive exercise with normal pre-exercise glycogen levels, depletion in muscle glycogen stores varies widely between participants and typically cannot be linked to muscle IL-6 or IL-8 mRNA expression (10, 11, 14–16). Moreover, data from our laboratory indicate that even though cycling induces greater vastus lateralis muscle glycogen depletion in comparison to running, muscle cytokine mRNA expression is multiple times greater with running (10, 11). IL-6 release is markedly higher from the arm compared with the leg during whole-body exercise, and is not linked to muscle glycogen content (15). Additionally, carbohydrate compared to placebo ingestion during prolonged and intensive exercise increases blood glucose levels and decreases muscle mRNA expression and plasma levels for IL-6 and IL-8 despite similar muscle glycogen depletion rates (10, 11). In the current study, subjects drank water *ad libitum* during the mid-afternoon running trials (3-h post-prandial state), without ingestion of any other beverages or food. Thus carbohydrate ingestion would have been expected to attenuate post-exercise increases in plasma IL-6 and IL-8, perhaps weakening the linkage to decreases in muscle glycogen content. Although the data are not entirely consistent, regular exercise bouts in the fasted state without oral carbohydrate intake may enhance training adaptations (32, 33).

This is the first study to simultaneously measure muscle mRNA expression, muscle protein content, and plasma levels for three cytokines (IL-6, IL-8, and MCP-1) after exhaustive exercise. Interpretation of the data from this study was strengthened by combining results from two intense, long duration exercise sessions for 24 male runners (28), in contrast to other studies utilizing small subject numbers and modest exercise workloads (14–17). While others have shown increases in skeletal muscle MCP-1 mRNA following exercise (8), this study demonstrated that post-exercise increases in both muscle MCP-1 mRNA expression and protein content are unrelated to post-exercise muscle glycogen levels. Della Gatta et al. (8) showed that muscle mRNA expression and protein content for IL-6, IL-8, and MCP-1 were increased in biopsy samples obtained from eight untrained men 2 h following intensive leg resistance exercise. Plasma cytokine levels were not reported in this study, but data suggested that MCP-1 and IL-8 were expressed by cells in the interstitial space between muscle fibers, including macrophages, satellite cells, and other stromal cells (8). A limitation in the current study is that repeated muscle and blood samples were not collected during the first several hours post-exercise that may have revealed additional insights.

Taken together, under the normal context within which athletes train and compete, muscle glycogen depletion at best represents just one potential signal for cytokine mRNA expression within muscle tissue. Other signaling mechanisms may include free fatty acid availability, blood glucose levels, nitric oxide, and exercise-induced muscle damage and adaptation (8, 11, 16, 20). Nonetheless, our data support a significant, modest, negative relationship of post-exercise muscle glycogen and plasma cytokine levels (13), and this could be due in part to the release of IL-6, IL-8, and MCP-1 from extra-muscular sources, including adipose tissue (3, 16) and the brain (7). Additionally, our data support a significant, negative relationship between post-exercise muscle glycogen content and both muscle and plasma IL-6 protein levels, but not muscle IL-6 mRNA expression, suggesting that unmeasured factors within the secretome of the working skeletal muscle may differentially influence cytokine mRNA stability, transcription, production, degradation, modification, and release into the circulation (12, 13).

## Conflict of Interest Statement

The authors declare that the research was conducted in the absence of any commercial or financial relationships that could be construed as a potential conflict of interest.

## Funding

Reoxcyn Discoveries Group, Salt Lake City, UT, USA.
